# Cancer-associated Fibroblast–specific Expression of the Matricellular Protein CCN1 Coordinates Neovascularization and Stroma Deposition in Melanoma Metastasis

**DOI:** 10.1158/2767-9764.CRC-23-0571

**Published:** 2024-02-27

**Authors:** James Hutchenreuther, John Nguyen, Katherine Quesnel, Krista M. Vincent, Louis Petitjean, Sophia Bourgeois, Mark Boyd, George Bou-Gharios, Lynne-Marie Postovit, Andrew Leask

**Affiliations:** 1 Department of Physiology and Pharmacology, University of Western Ontario, London, Ontario, Canada.; 2 College of Dentistry, University of Saskatchewan, Saskatoon, Saskatchewan, Canada.; 3 Department of Dentistry, University of Western Ontario, London, Ontario, Canada.; 4 Department of Oncology, University of Alberta, Edmonton, Alberta, Canada.; 5 Department of Medical Genetics, Children's Hospital of Eastern Ontario, Ottawa, Ontario, Canada.; 6 Pharmanest, Inc., Princeton, New Jersey.; 7 Department of Biology, University of Western Ontario, London, Ontario, Canada.; 8 Office of the Vice President of Research, University of Saskatchewan, Saskatoon, Saskatchewan, Canada.; 9 Department of Musculoskeletal and Ageing Science, Institute of Life Course and Medical Sciences, University of Liverpool, Liverpool, United Kingdom.; 10 Department of Biomedical and Molecular Sciences, Queens University, Kingston, Ontario, Canada.

## Abstract

Melanoma is the leading cause of skin cancer–related death. As prognosis of patients with melanoma remains problematic, identification of new therapeutic targets remains essential. Matricellular proteins are nonstructural extracellular matrix proteins. They are secreted into the tumor microenvironment to coordinate behavior among different cell types, yet their contribution to melanoma is underinvestigated. Examples of matricellular proteins include those comprising the CCN family. The CCN family member, CCN1, is highly proangiogenic. Herein, we show that, in human patients with melanoma, although found in several tumor cell types, CCN1 is highly expressed by a subset of cancer-associated fibroblasts (CAF) in patients with melanoma and this expression correlates positively with expression of proangiogenic genes and progressive disease/resistance to anti-PD1 checkpoint inhibitors. Consistent with these observations, in a syngeneic C57BL6 mouse model of melanoma, loss of CCN1 expression from Col1A2-Cre-, herein identified as “universal,” fibroblasts, impaired metastasis of subcutaneously injected B16F10 tumor cells to lung, concomitant with disrupted neovascularization and collagen organization. Disruption of the extracellular matrix in the loss of CCN1 was validated using a novel artificial intelligence–based image analysis platform that revealed significantly decreased phenotypic fibrosis and composite morphometric collagen scores. As drug resistance is linked to matrix deposition and neoangiogenesis, these data suggest that CCN1, due to its multifaceted role, may represent a novel therapeutic target for drug-resistant melanoma. Our data further emphasize the essential role that cancer-associated, (universal) Col1A2-Cre-fibroblasts and extracellular matrix remodeling play in coordinating behavior among different cell types within the tumor microenvironment.

**Significance:**

In human patients, the expression of proangiogenic matricellular protein CCN1 in CAFs correlates positively with expression of stroma and angiogenic markers and progressive disease/resistance to checkpoint inhibitor therapy. In an animal model, loss of CCN1 from CAFs impaired metastasis of melanoma cells, neovascularization, and collagen deposition, emphasizing that CAFs coordinate cellular behavior in a tumor microenvironment and that CCN1 may be a novel target.

## Introduction

Melanoma, the second most common invasive cancer in young adults, although accounting for only 4% of skin cancers, accounts for over 80% of skin cancer deaths due to its metastatic nature (1, 2). Melanoma, unlike other cancers, is largely unaffected by chemotherapies and radiotherapies (3); however, encouragingly, surgical excision is highly effective in nonmetastatic melanoma, with an approximately 90% 5-year survival rate (4, 5). However, if tumors metastasize, the survival rate is extremely poor (4, 5). Identification of novel drug therapies is therefore essential.

The recent identification of mutations resulting in constitutive activation of BRAF, a protein kinase that activates ERK, has led to the development of useful drugs that target late-stage melanoma (6, 7). As most (92%) of these mutations occur in the same codon, namely BRAFV600E (7, 8), several of these drugs target BRAFV600E. Unfortunately, these drugs show only an approximately 50% response rate, and only increase overall survival by approximately 4 months (8, 9). Moreover, patients rapidly acquire resistance to these drugs (9), in a fashion that appears to involve, at least in part, the deposition of a stiff extracellular matrix (ECM) network around the tumor through the action of cancer-associated fibroblasts (CAF) via the matrix stiffness–induced activation ERK through stimulation of the proadhesive integrin β1/focal adhesion kinase (FAK) pathway (10–12). Similarly, angiogenesis or immune checkpoint inhibitors (ICI) have been developed, but these have limitations in practice, as, for example, the fibrotic stroma can impair T-cell penetration into the tumor (13–18). Moreover, ICIs can also result in neoangiogenesis by stimulating hypoxia-inducible factor-1α (HIF1α; refs. 16–18). It has been hypothesized that the therapeutic efficiency of anticancer drugs could be enhanced by combinatorial treatment with antifibrotic or antiangiogenic drugs.

Matricellular proteins secreted by CAFs may represent suitable antifibrotic and antiangiogenic targets (19). CAFs not only secrete cytokines into the tumor microenvironment but also produce *de novo* a stiff ECM that surrounds the tumor (19). One such matricellular protein, the CCN family member CCN1 (Cyr61, cysteine-rich protein 61), is regulated by mechanotransduction via Yes-associated protein (YAP)/transcriptional co-activator with PDZ-binding motif (TAZ) and hypoxia via HIF1α and has multiple context-specific and cell type–specific effects most notably in promoting angiogenesis (including in the placenta), vascular integrity, and cardiac morphogenesis (20–24). CCN1’s proangiogenic activity occurs through integrin α_v_β_3_ (25, 26). Similarly, in human dermal fibroblasts, CCN1 activates adhesive signaling, including ERK activation, and induces a wound healing gene expression program through heparin sulfate-containing proteoglycans and integrin α6β1 (27, 28). Interestingly, we recently found that fibroblast-specific deletion of CCN1, although not appreciably affecting mechanical stiffness of skin or cutaneous tissue repair, resulted in impaired collagen filament organization and resistance to bleomycin-induced skin fibrosis (29). If fibroblast-specific expression of CCN1 is required for ECM elaboration in tumor stroma remains uninvestigated.

Although it promotes colorectal cancer, pancreatic, ovarian, and breast cancer (30), the role of CCN1 in patients with melanoma remains unclear. In this article, we use a syngeneic model of melanoma metastasis to begin to probe the role of the proangiogenic matricellular protein CCN1 in melanoma. Data were validated using publicly-available databases derived from patients with melanoma. Our data provide new and valuable insights into how fibroblast-specific expression of a matricellular protein coordinates activity among multiple cell types in the tumor microenvironment and suggest that CCN1 may represent a novel therapeutic target for drug-resistant melanoma metastasis.

## Materials and Methods

### Cell Culture

B16-F10 murine melanoma cell (ATCC catalog no. CRL-6475, RRID:CVCL_0159) was cultured in DMEM, 10% FBS, and 1% antibiotic/antimycotic solution (Invitrogen). CCN1-deficient mouse embryonic fibroblasts were isolated and cultured as described previously (29). Cells were not routinely tested for the presence of *Mycoplasma* but were authenticated using ATCC's Mouse short tandem repeat Profile service (99% match database profile).

### Animals and *in vivo* Tumor Implantation

All animal protocols were approved by the animal care committee at the University of Western Ontario or the University of Saskatchewan (Saskatoon, Saskatchewan, Canada). C57BL6J mice hemizygous for a tamoxifen-dependent Cre recombinase expressed under the control of a fibroblast-specific Col1a2 promoter/enhancer [Col1A2-Cre(ER)T)] that were also ccn1^f/f^ were created and genotyped as described previously (29). Please note Col1a2-Cre(ER)-T mice contain a fibroblast-specific far upstream enhancer, initially identified in the laboratory of Benoit de Crombrugghe, subcloned upstream of the Col1a2 minimal promoter; previous publications have extensively shown that this construct permits transgene expression only in fibroblasts and not in other collagen-expressing cells such as osteoblasts and chondrocytes (31). Tamoxifen, an estrogen inhibitor, has been reported to suppress estrogen-induced CCN1 expression in cancer cells (32). Therefore, to circumvent this confounding issue, only male mice were used in this study. Male mice (3 weeks old) were injected intraperitoneally with tamoxifen (10 mg/mL) or corn oil (vehicle control) every day for 5 days to generate mice deleted or not for CCN1. In this article, mice not deleted for ccn1 in fibroblasts are designated CCN1^f/f^ whereas mice deleted for ccn1 are designated CCN1^−/−^. At 6 weeks old, mice were subjected to syngeneic model of melanoma metastasis, using B16-F10 cells (ATCC catalog no. CRL-6475, RRID: CVCL_0159), as described previously (33–35). Cells were freshly purchased from ATCC and used between passages 3–5. After detection of a palpable tumor, tumor growth was evaluated for 14 days, after which mice were euthanized. Mice were then either processed for micro-computed tomography (micro-CT; see below), or tumors were harvested by either embedding in TissueTek O.C.T. compound (VWR) and frozen, or by fixation in 4% paraformaldehyde (PFA) prior to embedding in paraffin. Tumor size was measured using calipers [tumor volume (mm^3^) = 1/2 (length of longest tumor dimension) × (length of narrowest tumor dimension)^2^], and percentage increase in tumor size from the initial palpable tumor was calculated, as indicated previously (35).

### Histologic Analysis

Paraffin-embedded sections (6 µm) were cut on a microtome. CCN1 or YAP1 was stained by applying first an anti-CCN1 (1:250 dilution, EMD Millipore, ABC102, rabbit polyclonal) or anti-YAP/TAZ (1:250 dilution, Cell Signaling Technology, 8418S, rabbit monoclonal) and subsequently an anti-rabbit horseradish peroxidase (HRP)-labeled polymer (Dako, K4003) followed by application of 3,3ʹ-diaminobenzidine (DAB) chromogen (Dako, K3468) for 20 or 30 minutes, respectively. For staining of myofibroblasts, the Vector Laboratories ABC-HRP staining kit (PK-4001) was used as according to the manufacturer's instructions using a DAB enzyme substrate (SK-4105) for colorimetric development. Sections were incubated with an anti-α-SMA (Abcam, ab5694; 1:400, RRID:AB_2223021) or anti-CD31 (1:200 dilution, sc-376764 Santa Cruz Biotechnology) antibody, as indicated. Images were taken using Zeiss microscope and camera. The percentage of anti-α-SMA positive cells (detected by the presence of nuclei) and the percentage of stromal area occupied by vessels staining positively for CD31 was calculated using ImageJ (RRID:SCR_003070). A Student *t* test was performed for statistical analysis.

To detect metastatic foci, lung sections were stained with hematoxylin and eosin (Thermo Fisher Scientific). Six mice per group were and 3 nonserial sections per lung were examined. Skin sections were stained using Masson's trichrome or Picrosirius red to examine collagen content. Using ImageJ (RRID:SCR_003070), the percent of the stroma area stained with alanine blue was determined. Student *t* test was used to analyze data.

### Proliferating Cell Nuclear Antigen and Terminal Deoxynucleotidyl Transferase–mediated dUTP Nick End Labeling Assays

Paraffin-embedded sections (5 µm) were double stained with anti-proliferating cell nuclear antigen (PCNA; 1:50 dilution, Invitrogen PA5-16797, rabbit polyclonal) and anti-α-SMA (1:200 dilution, Invitrogen 14-9760-82, mouse monoclonal), subsequently with Rhodamine-goat anti-rabbit (1:1,000, Jackson Immunoresearch 111-025-144, RRID:AB_2337932) and 488-goat anti-mouse (1:1,000 dilution, Jackson Immunoresearch 115-545-166, RRID:AB_2338852), followed by mounting media containing 4′,6-diamidino-2-phenylindole (DAPI; Vector Laboratories).

To detect DNA double-strained breaks caused by apoptosis, a terminal deoxynucleotidyl transferase–mediated dUTP nick end labeling (TUNEL) Assay, employing HRP-DAB (Abcam, ab206386), was used according to the manufacturer's protocol. Briefly, the sections were deparaffinized and rehydrated. Subsequently, permeabilized with Proteinase K (1:100 dilution) for 20 minutes immediately positive control was generated by incubating in 1 µg/µL DNase I for 20 minutes while other sections incubated in 1X TBS. Endogenous peroxidase was inactivated with 3% H_2_O_2_ in methanol for 5 minutes. 3′ hydroxyl terminus of DNA break was labeled with biotin-tagged terminal deoxynucleotide transferase (TdT) for 1.5 hours and followed by streptavidin-HRP for 30 minutes; however, for negative control TdT was substituted with water. Chromogen DAB (3 minutes) was used for detection. Nuclei were counterstained using Methyl Green (3 minutes).

### Datasets

Single-cell RNA sequencing (scRNA-seq) data, derived from 6,879 melanoma tumor cells, were obtained from Gene Expression Omnibus (GEO; RRID:SCR_005012) GSE115978 in July 2019. Patient tumor RNA-seq (fragments per kilobase of transcript per million fragments mapped [FPKM]) and clinical data were obtained from GEO (RRID:SCR_005012) GSE78220 (36) in December 2020. Samples that did not have pretreatment RNA sequencing completed were excluded from downstream analyses. One patient had two pretreatment RNA-seq samples; for this patient, the two CAF-specific CCN1 scores were averaged, and this average was included in downstream analyses. Level 3 The Cancer Genome Atlas (TCGA) RNAseqV2 gene expression was obtained from TCGA Data Portal in January 2016. Data were analyzed as described previously (34). Stromal scores were defined for tumors using the ESTIMATE [Estimation of STromal and Immune cells in MAlignant Tumor tissues using Expression data; original publication (37)] algorithm using RNASeqV2 data.

### CAF-specific *CCN1* Scores

CAF-specific *CCN1* scores were calculated for tumors by summing Z-scores for genes that were: (i) strongly correlated with *CCN1* (Pearson *r* > 0.5) in CAF single cells, (ii) had 10-fold higher mean expression in CAFs than any other cell type, and (iii) had mean expression ≤0.1 TPM/10 in malignant cells. This resulted in a six-gene score composed of the following genes: *PDGFRA*, *COL1A1*, *DCN*, *TAGLN*, *COL6A3*, and *LPAR1*.

### Gene Set Enrichment Analysis

Gene sets correlated with CAF-specific *CCN1* scores were determined by using Generally Applicable Gene-set Enrichment (GAGE, v2.12.3). Hallmark gene sets were downloaded from the Molecular Signatures Database (http://software.broadinstitute.org/gsea/msigdb) v5.0 on August 10, 2015. Enrichment was calculated against a formulated sample composed of the mean expression values for each gene and sample-specific test statistics were correlated to CAF-specific *CCN1* scores using Spearman rank correlation (38).

### micro-CT Analysis

Mice were given intraperitoneal sodium heparin to prevent coagulation, and then euthanized with isoflurane asphyxiation. A butterfly needle was inserted into the left ventricle and heparinized saline was run through it at approximately 120 mmHg to flush the blood from the vasculature. A small incision in the right ventricle allowed drainage. Mice were then perfused with Microfil (Flow-Tech), which was allowed to polymerize for approximately 30 minutes before the mice were fixed in PFA (∼24 hours). After preliminary CT scans to ensure full perfusion tumors were removed and embedded in paraffin for high-resolution X-ray micro-CT on a GE Locus MS-8 X-ray conebeam micro-CT imaging system (GE Medical Systems). micro-CT imaging system was operating at a peak energy of 80 kVp and tube current of 80 µA, acquiring 900 X-ray projections (average four frames with integration time of 3,000 ms) at 0.4-degree angular increments during a full 360-degree rotation of the sample. Visualization and analysis of the three-dimensional micro-CT data was performed in MicroView (GE Healthcare, Parallax Innovations).

### Survival Analysis

The association between CAF-specific CCN1 score and overall survival in the GSE78220 cohort (36) was tested in multivariate Cox regression models with CAF-specific CCN1 score considered as a continuous variable. In the multivariate setting, adjustment was conducted using the following risk factors: age, gender, previous MAPK inhibitor treatment, BRAF mutation positivity, NRAS mutation positivity, and NF1 mutation positivity. All analyses and visualizations were conducted in the RStudio programming environment version 2021.09.2 (RStudio Inc., RRID:SCR_000432). R/Bioconductor packages ggplot2, gplots, survival, GAGE, and plyr were used, where appropriate. Z-score unsupervised hierarchical clustering was conducted using 1-c (where c is the Pearson correlation coefficient) as the distance and the Ward agglomeration method (ward.D2). Statistical analysis was conducted using unpaired *t* test. RECIST response was defined as per the original article (36): Responding pretreatment tumors were derived from patients who went on to have complete or partial responses or stable disease control (with mixed responses excluded) in response to anti-PD-1 therapy. Nonresponding tumors were derived from patients who had progressive disease. These response patterns were based on Immune-related Response Evaluation Criteria In Solid Tumors (irRECIST) (39, 40).

### Real-time PCR Analysis

RNA isolated, using TRIzol, from fibroblasts cultured from three independent mice were used for each datapoint. Alternatively, tissue was homogenized in 1 mL of TRIzol and a TRIzol-chloroform extraction was performed. Cells were used at passage 5. TAQman PCR was performed on 40 ng of mRNA to examine expression of Ccn1 (Mm00487498_m1) and Mmp9 (Hs00957562_m1). Experiments were run in triplicate (Applied Biosystems, RRID:SCR_018060), delta-delta CT method was used for analysis using 18S RNA as an internal control. Statistical analysis was conducted using unpaired *t* test (*N* = 3). Tissue samples were run in parallel to those published previously (29).

### Flow Cytometry

Tumors, maintained in 1X DPBS + 5% FBS, were excised, minced, and digested for 20–24 hours at 4°C on a vertical shaker in 1X DMEM, 10% FBS, 0.5 mg/mL Collagenase P (Sigma, 11213857001), 10 µg/mL DNase I (Sigma, D5025). Tissue was filtered through a 50 µm strainer and washed with full media (1X DMEM + 10% FBS) and centrifuged for 5 minutes at 450 × *g* to obtain a cell pellet. This process was repeated twice. Red blood cells (RBC) were lysed using 1 mL of lysis buffer (Invitrogen, 00-4333-57) for 1 minute, followed by addition of 9 mL full media, and centrifugation for 5 minutes at 450 × *g*. The resultant cell pellet was resuspended in 1XDPBS + 5% FBS, and a single-cell suspension was achieved by filtering the resuspended cell pellet through a 40 µm strainer.

A total of 1 × 10^6^ cells were added to each well of a 96-well plate. Cell pellets were obtained by centrifuging for 5 minutes at 450 × *g* and washing once with 1X DPBS + 5% FBS and were subsequently resuspended in Fc receptor blocking buffer; 10% Fc blocking reagent (Miltenyi Biotec, 130-092-575) in 1X DPBS + 5% FBS for 1 hour on ice, in the dark. Cells were then incubated (1 hour, on ice, in the dark) with 1 µL of each antibody per 1 × 10^6^ cells [CD3 eFluor 506 (Invitrogen, 69-0032-80), CD45 NovaFluor-Red 710 (Invitrogen, M005T02R04), CD4 PE-Texas Red (Invitrogen, MCD0417), CD8 FITC (BioLegend, 140403)]. After this incubation step, cells were pelleted (5 minutes, 450 × *g*) washed twice and resuspended in 1X DPBS + 5% FBS.

Flow cytometry was performed on a Cytoflex Multicolor Flow equipment (Beckman Coulter, RRID:SCR_019627). CD3, CD45, CD4, and CD8 markers were detected using Violet channel KO525 (525/40), Red channel APC-A700 (712/25), Blue channel ECD (610/20), and Blue channel FITC (525/40), respectively. Data were obtained and analyzed using CytExpert v2.5 (Beckman Coulter; RRID:SCR_017217).

### Histologic Image Analysis

The histologic glass slides were imaged and photographed using an Olympus CK53 microscope and DP23 camera at 40X (∼0.22 mm/pixel). The digital image files were uploaded to the FibroNest (PharmaNest Inc) digital pathology platform for analysis. After color normalization and standardization (41), the image was automatically processed to remove anomalies such as scanning stripes, image compression artifacts, rinsing artefacts, dusts, and saturated pixels. The digital image is then processed and segmented to allocate the collagen biological marker to a specific channel. The full tissue is automatically detected, and the analysis region of interest (ROI) is defined 50 mm away from the edge of the tissue to avoid edge artifacts. The single-fiber artificial intelligence digital pathology platform, FibroNest, extensively characterizes both absolute collagen content and important statistical features of the distributions of collagen fibers’ morphometric and architectural phenotypes. More specifically, the software analyses each collagen fiber to quantify histologic traits that reflect collagen context (12 traits), collagen fiber morphometry (13 traits), and fibrosis architecture (seven traits; refs. 42–45).

Fibers are classified in two classes (Fine, Assembled) based on the complexity of their structure (defined as morphometric skeleton) and related to the progression and reticulation of fibers. Histograms reflecting the statistical distribution of each trait across biopsies were evaluated further to determine a variety of statistical values such as mean, median, and SD, skewness and kurtosis (these parameters are sensitive to the distortion of the histograms). For each trait and its related histogram, cut-off values are selected to identify and quantify the presented of fibrosis severity in the histograms. These seven statistical values are continuous variables defined as quantitative fibrosis parameter trait (qFT). qFTs are also normalized for area to be independent of the selection of the ROIs. The qFTs that best account for the progression of fibrosis severity have been identified in prior studies (46) and combined to form a phenotypic fibrosis composite score (Ph-FCS) which is an overarching continuous quantification of the severity fibrosis phenotype (47). Similarly, the qFTS that specifically relate to the morphometry of the fibers are normalized and assembled in a morphometric composite score. The very high detection threshold of the method was described and used in preclinical studies (48, 49).

### scRNA-seq Analysis

Col1A2-Cre(ER)T/0; ROSA26mTmG mice (#007576, Jackson Laboratories RRID:IMSR_JAX:007576) were used to specifically label Col1A2-Cre-fibroblasts with GFP. At 3 weeks old, mice were treated with tamoxifen (10 mg/mL) or corn oil (vehicle control) every day for 5 days. Six weeks later, mice were euthanized and skin collected. Skin was then digested with 2 mg/mL Collagenase type IV (Life Technologies, 17104019) for 3 hours at 37°C. Digested skin was smashed and filtered through a 70 µm strainer. Cells were pelleted (5 minutes, 450 × *g*) and resuspended in RBC lysis buffer (Invitrogen, 00-4333-57) for 3 minutes before being pelleted (5 minutes, 450 × *g*). Finally, cells were brought into single-cell suspension for FACS by filtering through a 40 µm strainer. GFP-tagged Col1A2-Cre-fibroblasts were then isolated using BD FACSMelody (BD Biosciences) and a 488 nm blue laser with a BP/527/32/LP/507/5 filter. Library construction, sequencing, and data processing were performed by the Next Generation Sequencing Facility (University of Saskatchewan, Saskatoon, Saskatchewan, Canada), according to the manufacturer's instructions (Chromium iX, 10X Genomics). Approximately 6,000 cells, pooled from 3 different mice, were sequenced. RNA-seq data were examined using Loupe Browser v.6.5.0 (10X Genomics) and are available at GEO253482. We defined Defb8+, Crabp1+ as papillary dermal fibroblasts, Pi16+, Col15a1+, C3+, Cd84+ for universal dermal fibroblasts and Nexn, Trim63, Actn2, Hspb7 for reticular dermal fibroblasts, as described previously (50–54).

### Data Availability

Data and noncommercially available mice can be provided upon reasonable request or are at GEO253482.

## Results

### CCN1 Expression, in Patients with Melanoma, is Abundant in CAFs and Correlates with Expression of Proangiogeneic Genes

**FIGURE 1:**
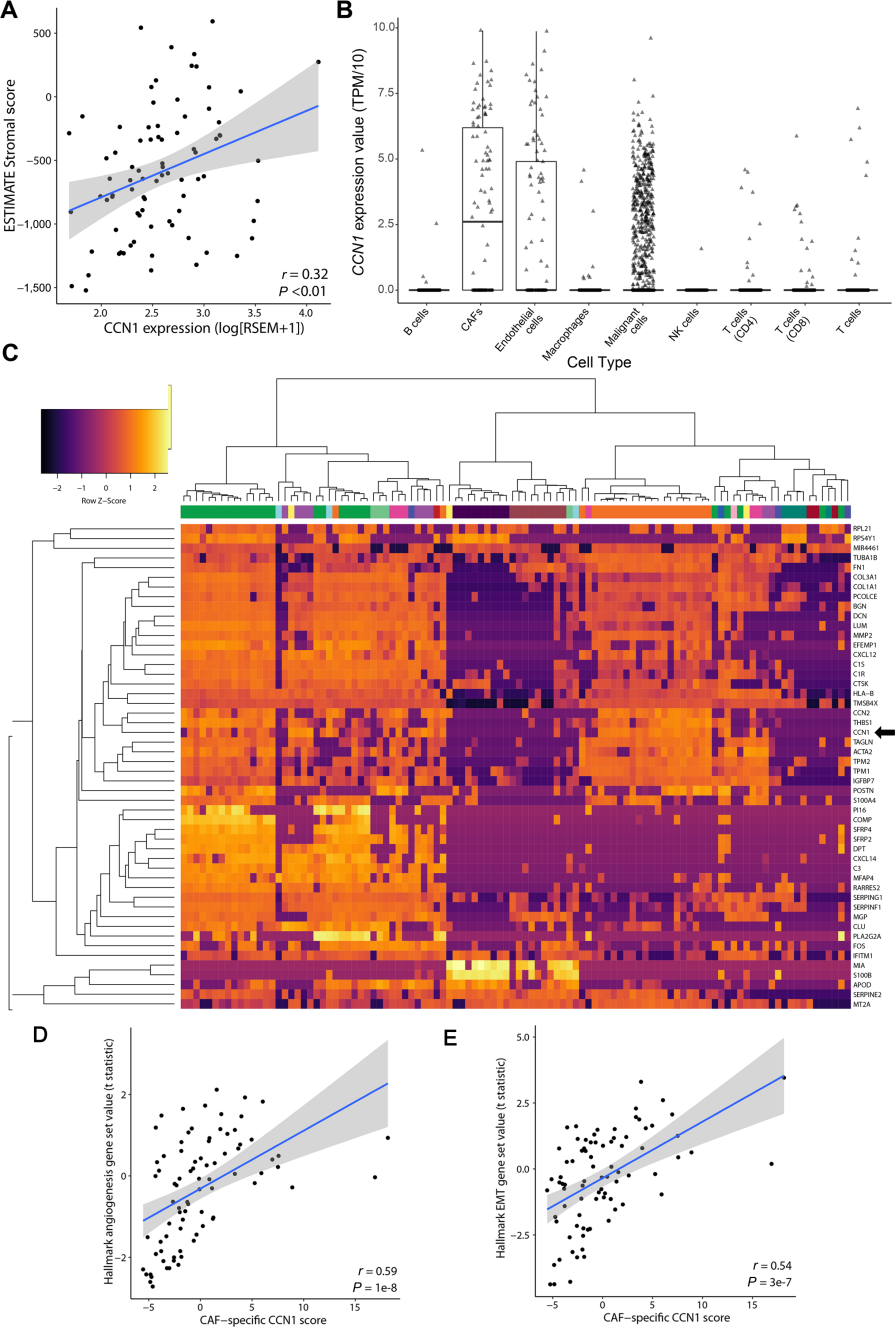
*CCN1* is expressed by a subset of CAFs and correlates with expression of angiogenic markers. **A,***CCN1* expression correlates with bulk tumor stromal scores. Scatterplot representation of stromal score (ESTIMATE algorithm) to *CCN1* expression in bulk primary melanoma tumors from TCGA (*n* = 82). The linear regression line is shown in blue, and the 95% confidence interval is shown as a gray area around the regression line. Spearman correlation coefficient and corresponding *P* value are displayed in the bottom right corner. **B,***CCN1* gene expression (TPM/10) in melanoma tumor single cells [malignant cells (*n* = 2,018), T cells (*n* = 3,321), B cells (*n* = 818), macrophages (*n* = 420), natural killer cells (*n* = 92), endothelial cells (*n* = 104), and CAFs (*n* = 106)]. Boxes represent interquartile ranges, and points represent individual sample values. **C,** Heat map representation of the 50 genes with the highest SD in CAF single cells from human melanoma tumors. CCN1 expression is higher in a particular subset of CAFs (GSE115978). Columns represent single CAF cells, and rows represent Z-score mRNA expression (TPM/10). The horizontal bar denotes different melanoma samples. Scatterplots show CAF-specific CCN1 scores versus gene set enrichment of angiogenesis (**D**), or EMT gene sets (**E**; GAGE *t* statistics) of melanoma bulk primary melanoma tumors from TCGA samples (*n* = 82). The linear regression line is shown in blue, and the 95% confidence interval is shown as a gray area around the regression line. Spearman correlation coefficient and associated *P* values are displayed in the bottom right corner.

Using melanoma bulk tumor RNA-seq data, we determined that *CCN1* expression correlated with stromal gene expression scores (Fig. 1A). Analysis of single-cell expression data revealed that, in patients with melanoma, *CCN1* was the most highly expressed in CAFs, but was also found in endothelial cells and malignant cancer cells (Fig. 1B). CAF expression of CCN1 was not ubiquitous, but clustered within a subset of CAF cells (Fig. 1C). To begin to determine what might be the role of CCN1 expression by CAFs, we used CAF single-cell expression data to identify a group of six genes (*PDGFRA*, *COL1A1*, *DCN*, *TAGLN*, *COL6A3*, and *LPAR1*) that strongly correlated with *CCN1* expression, yet were minimally expressed in other tumor cell types. The expression of these six genes was used as a surrogate score for CAF-specific *CCN1* expression in bulk tumor expression data (method similar to refs. 34, 38). To investigate possible mechanistic links, CAF-specific *CCN1* score was correlated with enrichment values (via sample-specific test statistics) of 50 hallmark gene sets in 389 melanoma patient samples. Top correlated gene sets include angiogenesis and epithelial-to-mesenchymal transition (Fig. 1D and E). These data suggested the hypothesis that CAF-specific expression of CCN1 may contribute to melanoma progression. Thus, we elected to evaluate, using a genetic model, the contribution of CAF-specific expression to melanoma metastasis.

### Universal Fibroblast-specific Deletion of *Ccn1* Impairs Metastasis of Melanoma Cells to the Lung, Concomitant with Impaired Collagen Organization and Neovascularization

**FIGURE 2:**
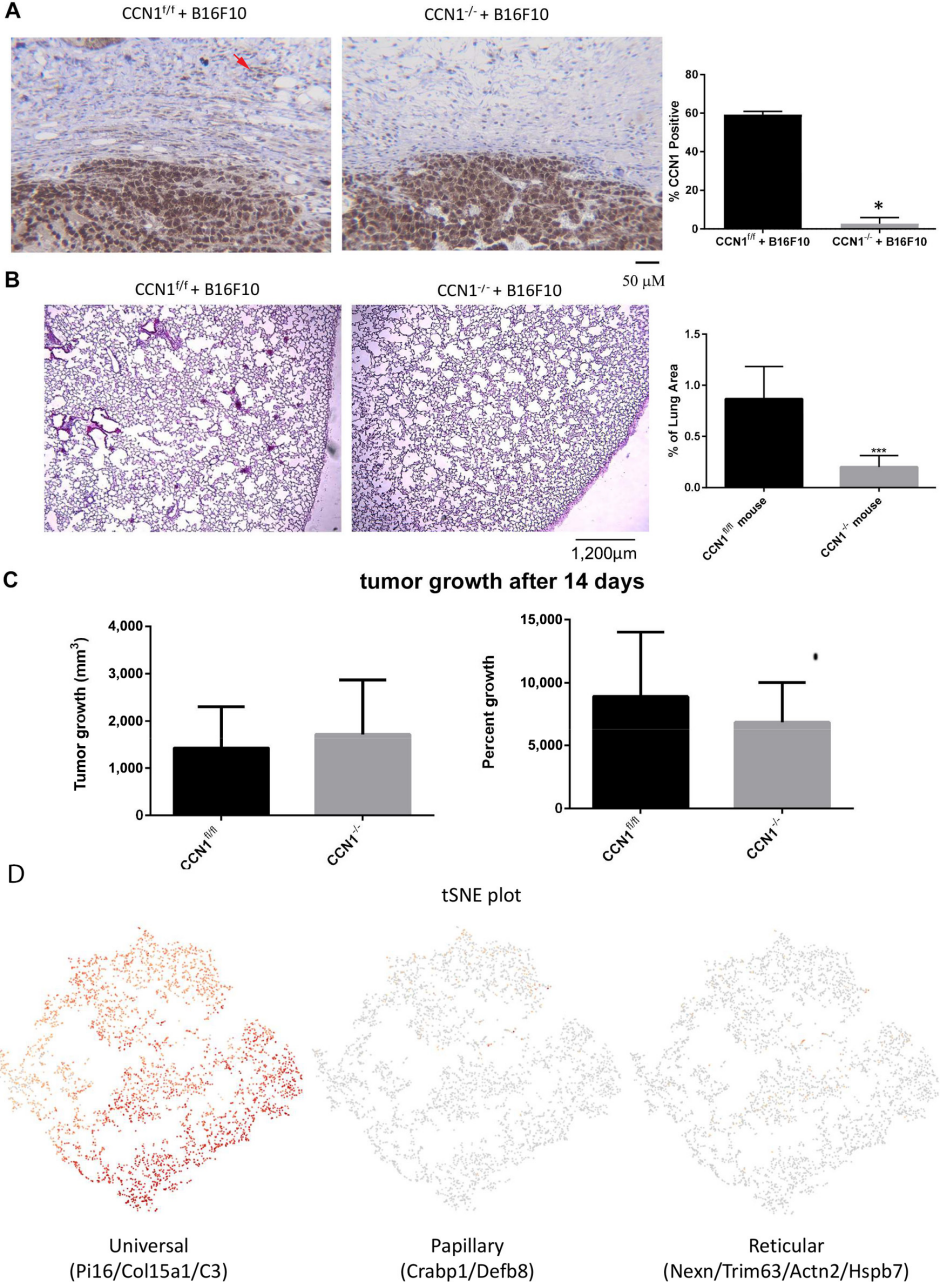
Mice harboring Col1A2-Cre-fibroblast–specific deletion for *Ccn1* show impaired metastasis of B16F10 melanoma cells to the lung. **A–C,** Mice were examined after 14 days of tumor growth. **A,** Sections of tumor stroma were stained with anti-CCN1 antibody (*N* = 3, representative images are shown), verifying loss of CCN1 protein expression in mice deleted for CCN1 in fibroblasts. Note CCN1 staining remains in the tumor. Scale bar = 50 µm. **B,** Representative images of lung sections from wild-type or mice harboring a deletion for *Ccn1* in Col1A2-Cre-fibroblasts. Hematoxylin and eosin was used to reveal detect dense metastatic foci (purple). Scale Bar = 300 µm. Total area of the lung section covered by metastases was quantified. Deletion of *Ccn1* caused reduced metastasis (*N* = 6, ***, *P* < 0.001). **C,** Reduced CCN2 expression in the stroma did not significantly alter tumor growth. Kruskal–Wallis analysis (*N* = 8). **D,** To more accurately define the cells in which CCN1 was deleted, three-week old Col1A2-cre(ER)T/); mTmG mice were injected for 5 consecutive days with tamoxifen to activate Cre recombinase. Six weeks later, skin was isolated, digested with collagenase, and cells expressing GFP were isolated by FACS, and subjected to RNA-seq analysis. Col1A2 lineage fibroblasts were identified as universal fibroblasts based on the gene expression of Pi16, Col15a1, C3, and Cd84 genes, but not papillary or reticular fibroblasts based on the gene expression of Crabp1 and Defb8 or Nexn, Trim63, Actn2, and Hspb7, respectively.

To examine the role of Col1A2-Cre-CAF–specific *Ccn1* expression in melanoma metastasis, we used mice expressing a tamoxifen-dependent Cre recombinase expressed under the control of a fibroblast-specific promoter/enhancer that was derived from the human *COL1A2* gene (29). Mice were also homozygous for a floxed-CCN1 allele. These mice were used to delete *Ccn1* in Col1A2-Cre-fibroblasts 3 weeks postnatally. Two weeks later, mice were subcutaneously injected with the poorly immunogenic B16F10 melanoma cell line (33–35). After detection of a palpable tumor, tumors were allowed to grow for an additional 14 days, after which animals were sacrificed, and tissue harvested. Deletion of *Ccn1* in Col1A2-Cre-CAFs (i.e., universal fibroblasts) was verified using an anti-CCN1 antibody to stain the resultant tissue sections (Fig. 2B). Note that CCN1 was still expressed normally in melanoma cells (Fig. 2A). We found that mice harboring a fibroblast-specific deletion of *Ccn1* displayed significantly impaired metastasis of tumor cells to the lung (0.967% ± 0.297% vs. 0.247% ± 0.070%, CCN1^f/f^ vs. CCN1^−/−^, Fig. 2B). Conversely, loss of *Ccn1* expression did not appreciably affect tumor growth (Fig. 2C).

To more precisely clarify the fibroblast subset in which CCN1 was deleted, Col1A2-Cre(ER)-T/0; mT/mG mice were injected with tamoxifen to activate Cre at 3 weeks of age, and cells expressing GFP (i.e., cells in which the Col1A2 promoter/enhancer, initially defined by Bou-Gharios (31), was active at 3 weeks of age) were isolated using FACS analysis from skin 6 weeks later. FACS-sorted GFP-positive cells (∼6,000 pooled from 3 different mice) were subjected to scRNA-seq analysis, which revealed that the Col1a2 promoter was activated in so-called universal fibroblasts (Fig. 2D).

**FIGURE 3:**
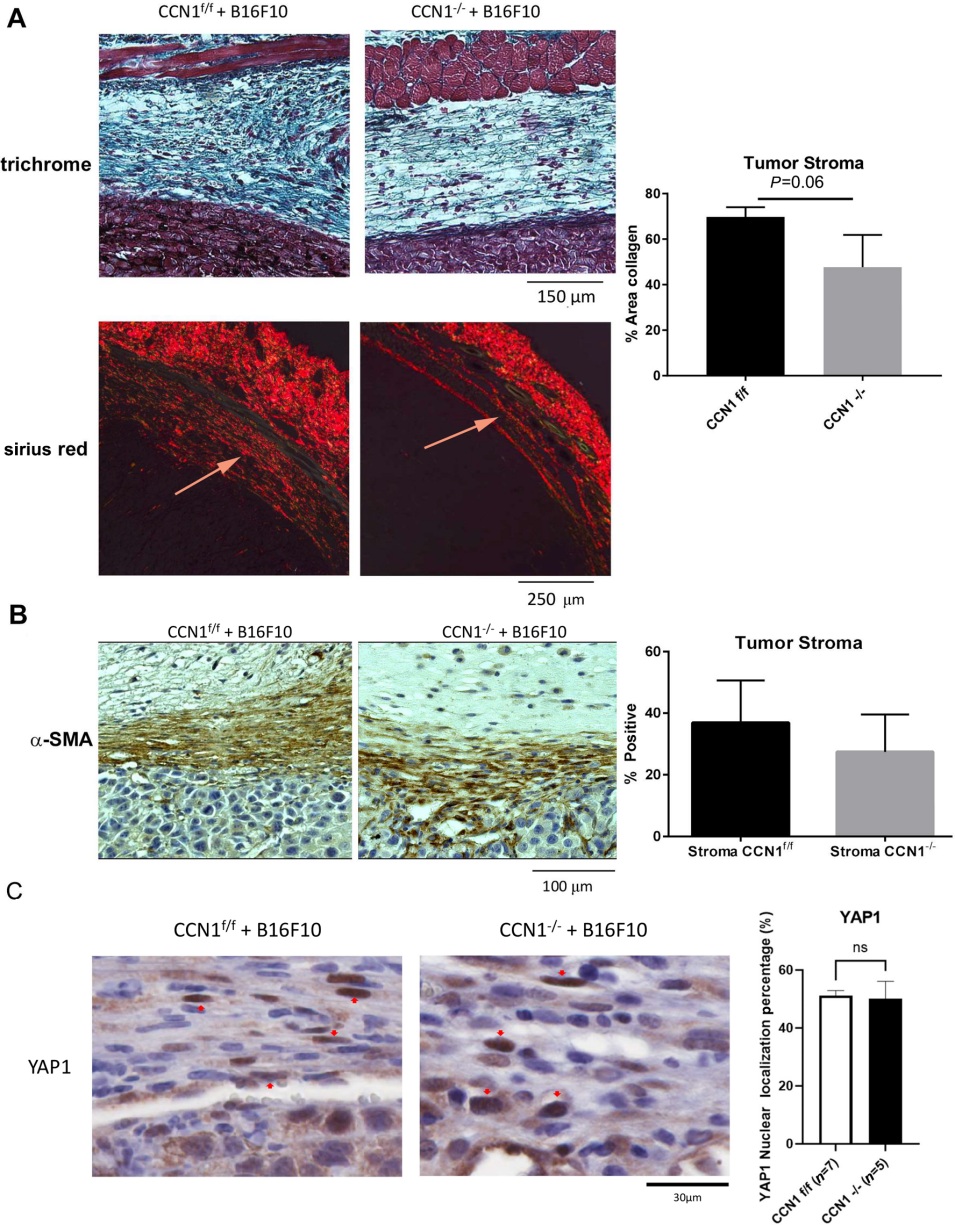
In a syngeneic model of metastasis, CCN1-deficient stroma possesses reduced collagen deposition, whereas myofibroblast formation is unaffected. Skin tissue showed a significant decrease in collagen shown trichrome and Sirius red staining (**A**; arrow indicates stroma). Representative images shown; Graph shows mean area of collagen, detected using trichrome stain, present in stroma images ± SD; Student *t* test; *n*  =  3). α-SMA–expressing myofibroblasts (**B**) and YAP-positive nuclei (**C**) are present in both wild-type and CCN1-deficient stroma (Representative images shown; Graphs represent mean α-SMA positive cells ± SD; one-way ANOVA; *n*  =  5). Mice were examined after 14 days of tumor growth of B16F10 melanoma cells.

**FIGURE 4:**
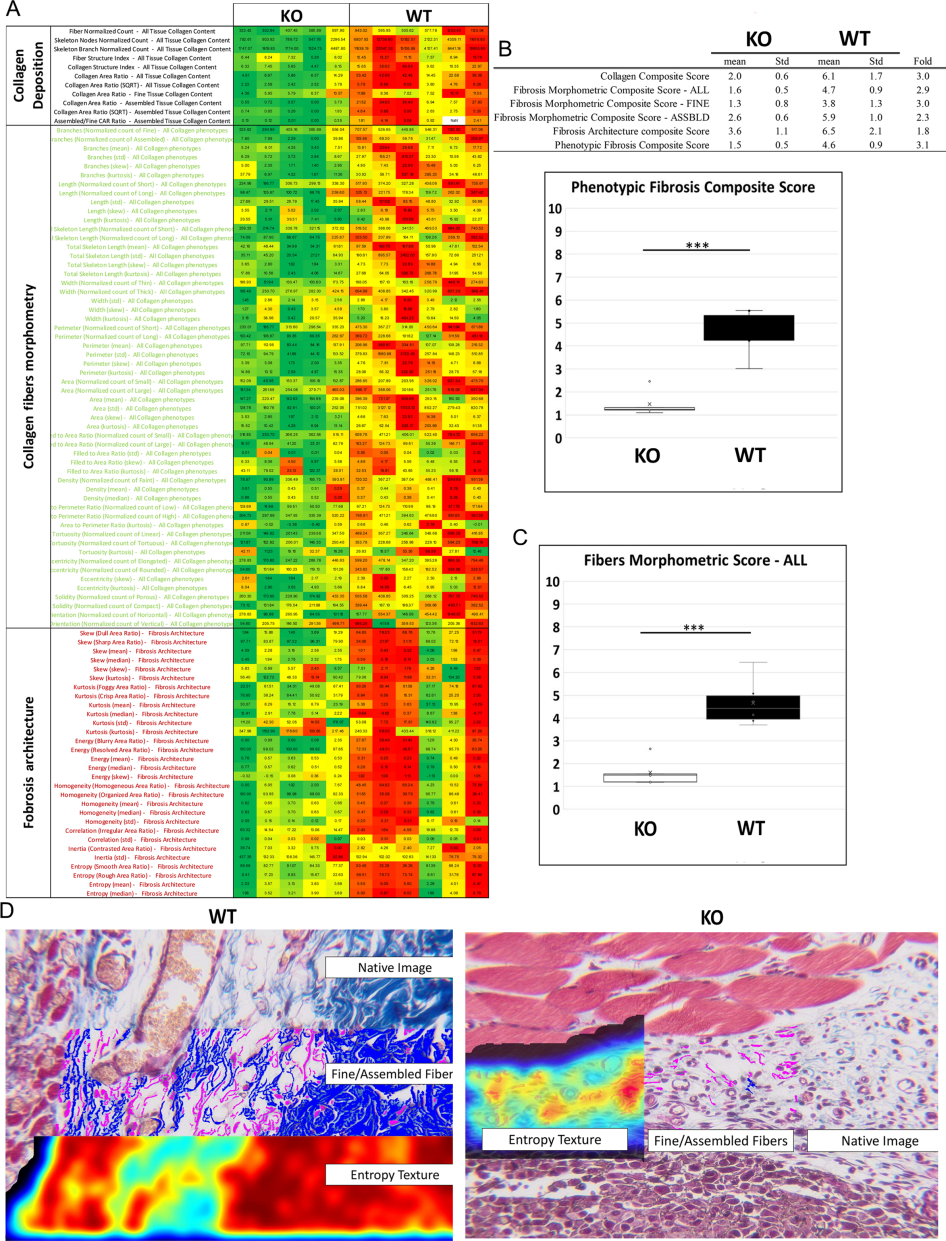
CCN1-deficient stroma show disorganized collagen fibers. Digital pathology phenotypic quantification (PharmaNest, Inc) phenotypic fibrosis histologic heat chart (**A**) where each row represents a principal qFT, at the three phenotypic levels (collagen deposition, fibers morphometry, and fibrosis architecture). **B,** The Ph-FCS recapitulates all the qFTs for one sample, and quantifies the phenotype of fibrosis and its differences between CCN1-deficient (KO; *n* = 5, mean = 1.5, Std dev = 0.5) and wild-type (WT) mice (*n* = 6, mean = 4.6, Std dev = 0.9) groups (3.1 fold change, *P* < 0.001, Student *t* test). **C,** The collagen fiber morphometric scores recapitulates the differences at the morphometric level between KO (*n* = 5, mean = 1.6, Std dev = 0.5) and WT (*n* = 6, mean = 4.5, Std dev = 0.9) groups (2.9 fold change, *P* < 0.001, Student *t* test). **D,** Representative images with augmented digital pathology layers. “Entropy Texture” is one of the architectural phenotypes of fibrosis (39). KO, knockout.

**FIGURE 5:**
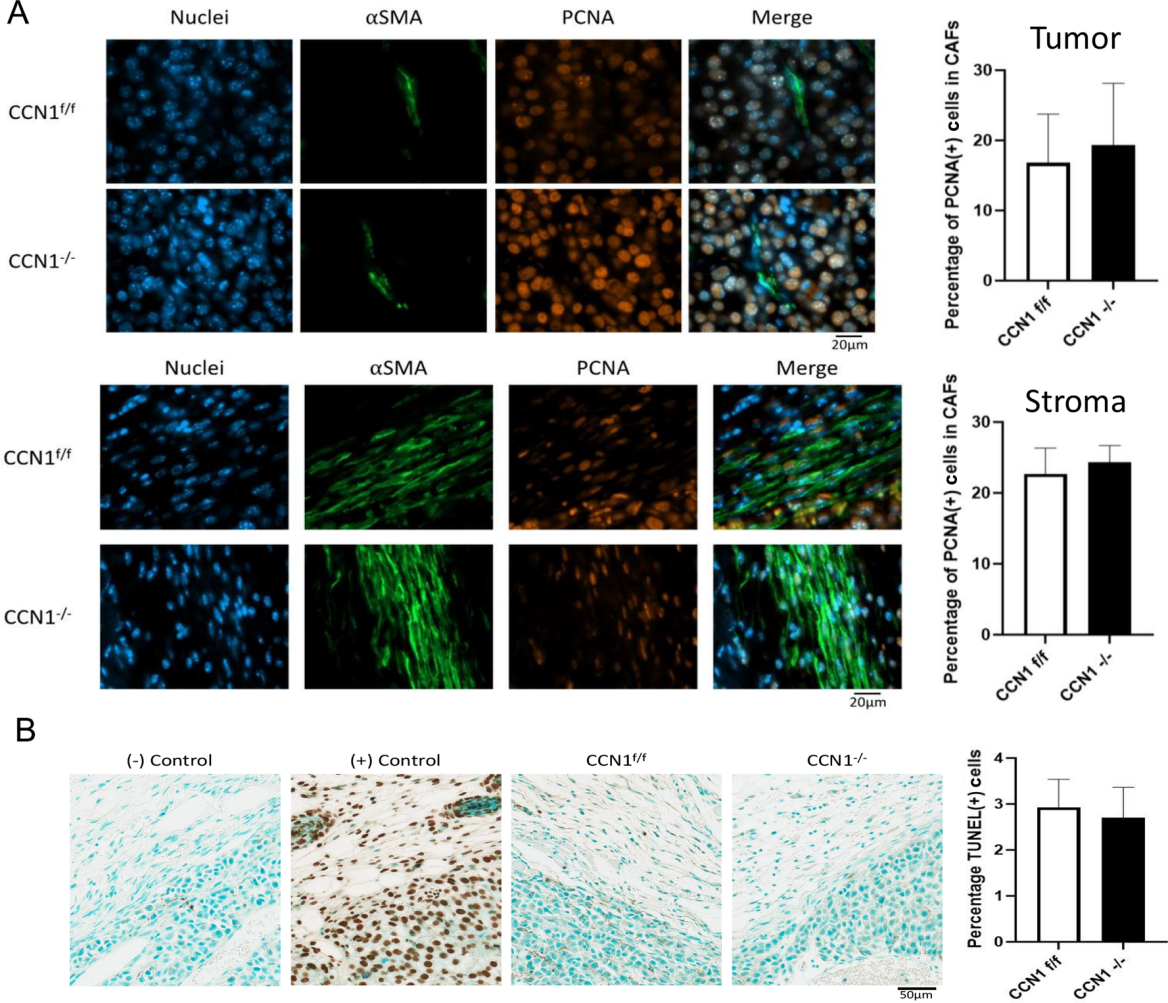
Loss of CCN1 expression from CAFs does not affect cell proliferation or apoptosis in a syngeneic model of melanoma metastasis. **A,** PCNA assay in (top) tumor and (bottom) stroma. Cell proliferation was detected using an anti-PCNA antibody. Representative images shown; graphs show the mean percentage of PCNA positive cells in CAFs ± SEM; CCN1^f/f^*n* = 7 and CCN1^−/−^*n* = 4, Student t test. **B,** TUNEL assay to detect apoptosis in stroma. Representative images shown; graphs show the mean percentage of TUNEL positive cells in stroma ± SEM, CCN1^f/f^*n* = 7 and CCN1^−/−^*n* = 4, Student *t* test.

CCN1-deficient stroma impaired tumor-associated collagen production (69.76% ± 2.44% vs. 47.65% ± 8.21%, CCN1^f/f^ vs. CCN1^−/−^, Fig. 3A). It was interesting to note that detailed image analysis of CCN1-deficient skin revealed that, compared with wild-type mice, mice deleted for *Ccn1* in Col1A2-Cre-CAFs displayed a nonlinear organization of collagen and discernable gaps in the collagen structure (Fig. 4). Myofibroblast differentiation, YAP nuclear localization (Fig. 3B and C), proliferation (as revealed by staining with an anti-PCNA) or apoptosis [using a Terminal deoxynucleotidyl transferase dUTP nick end labeling (TUNEL) assay] in tumors and stroma (Fig. 5A and B) were unaffected. These results extend prior data showing that fibroblast-specific expression of *Ccn1* was required for matrix remodeling, but not myofibroblast differentiation, in bleomycin-induced skin fibrosis (29).

**FIGURE 6:**
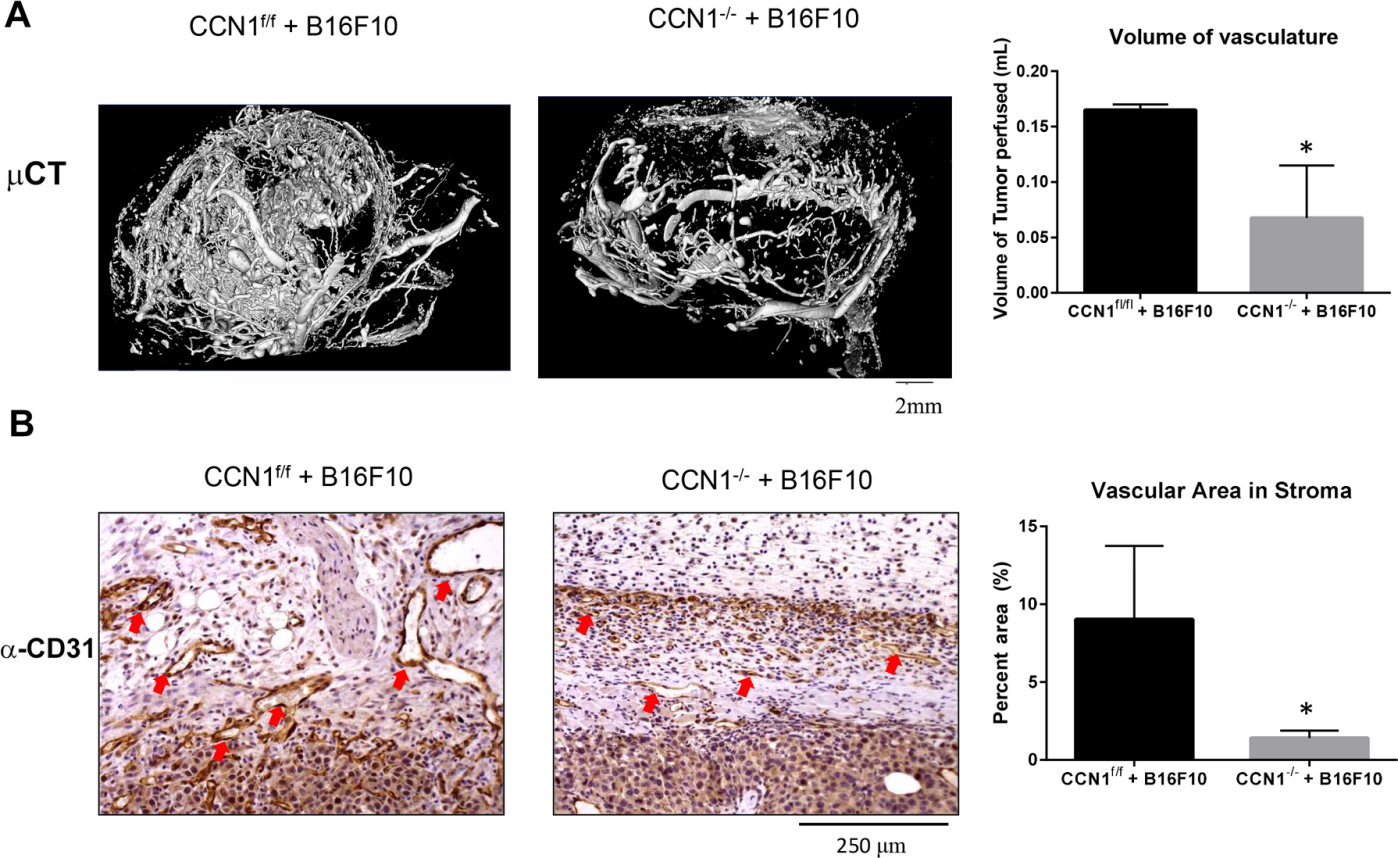
Deletion of *Ccn1* in Col1A2-Cre-fibroblasts reduces tumor vasculature. **A,** Tumors in wild-type or mice deleted for *Ccn1* in Col1A2-Cre-fibroblasts were perfused with a CT-contrast agent, as described in Materials and Methods. Volume (mL) of tumor occupied by vasculature was calculated. Mice lacking *Ccn1* in their fibroblasts had significantly reduced vascular volume (*t* test, *n* = 3, *P* < 0.05). **B,** Tumors and associated stroma in wild-type or mice deleted for *Ccn1* in fibroblasts were stained with anti-CD31 antibodies (*t* test; *n* = 3; *, *P* < 0.05).

Consistent with the previous observations that CCN1 induced angiogenesis via integrin α_v_β_3,_ (25), loss of fibroblast-specific expression of *CCN1* resulted in impaired neovascularization (0.165 ± 0.005 mL vs. 0.0675 ± 0.047 mL, CCN1^f/f^ vs. CCN1^−/−^), as detected by micro-CT analysis (Fig. 6A; see Supplementary Videos S1–S6). Results were substantiated by histologic analysis tumor stroma with an anti-CD31 antibody (Fig. 6B).

### CCN1 Expression in Fibroblasts is Associated with Negative Clinical Outcome

**FIGURE 7:**
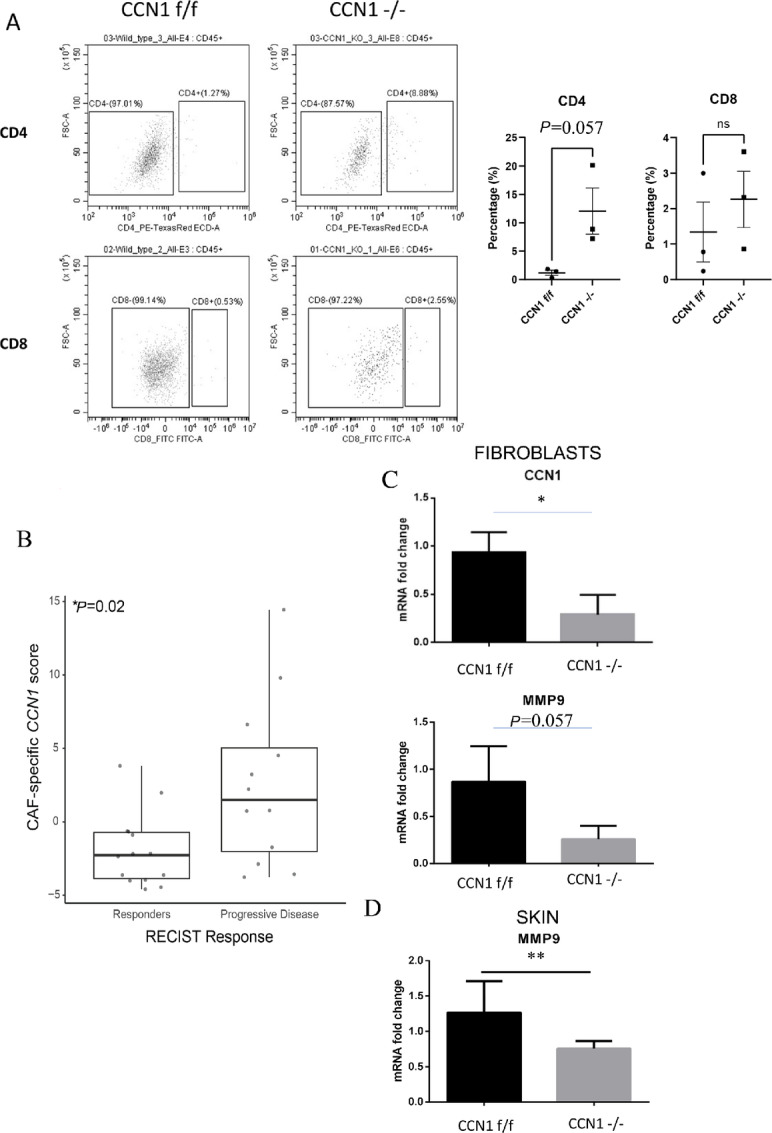
CCN1 expression by fibroblasts is associated with increased tumor penetration of CD4^+^ T cells and resistance to anti-PD1 checkpoint inhibitors. **A,** Tumors were subjected to flow cytometry with anti-CD4 anti-CD8 antibodies. Percentage of positive CD45-staining cells are indicated wild type (*n* = 3, CCN1^f/f^) and CCN1-deficient (*n* = 3, CCN1^−/−^) tumors (Student *t* test). Primary flow data of the median value is presented Tissue was examined after 14 days of tumor growth. **B,** Boxplot shows higher levels of CAF-specific *CCN1* scores in patients on checkpoint inhibitors with progressive disease (GSE78220). A six-gene set of CAF-specific genes correlating with *CCN1* (*PDGFRA*, *COL1A1*, *DCN*, *TAGLN*, *COL6A3*, and *LPAR1*), as described in Materials and Methods, was analyzed. *P* value (unpaired *t* test) displayed in top left corner. *n* = 14 in the responders and *n* = 12 in the progressive disease. **C** and **D,** Loss of *CCN1* expression results in reduced MMP-9 expression in CCN1-deficient mice. Real-time PCR analysis of RNAs isolated from *Ccn1^+/+^* and *Ccn1^−^^/^^−^* dermal fibroblasts from 3 different mice (*N* = 3; C) or skin (*N* = 4 *Ccn1^+/+^*, *N* = 8, *Ccn1^−^^/^^−^*; D) reveal that CCN1-deficient fibroblasts have reduced expression of the anti-PD1 resistance marker/effector MMP-9 (Student unpaired *t* test; *, *P* < 0.05). Please note that D was conducted on identical skin samples to those published previously (29).

Solid tumors can facilitate immune evasion by restricting T-cell migration; antifibrotic and antiangiogenic agents are likely to facilitate patient responsiveness to immunotherapy (55). As fibroblast-specific deletion of *Ccn1* resulted in impaired collagen organization and neovascularization, we reasoned that CCN1-deficient stroma would enhance the ability of CD4^+^ T cells to penetrate tumor cells. Indeed, flow cytometry with anti-CD4 antibodies revealed that, whereas few T cells penetrated the tumors embedded within a wild-type tumor stroma, there was a trend toward more CD4^+^ T cells (*P* = 0.057) being observable in CCN1-deficient stroma (1.20% ± 0.45% vs. 12.07% ± 4.06%, CCN1^f/f^ vs. CCN1^−/−^, Fig. 7A). Note that in both experimental groups few T cells were detected in the tumor is consistent with the notion that B16F10 tumors are poorly immunogenic (56–58), and that loss of *CCN1* expression in fibroblasts does not result in decreased tumor size (this article). *CCN1* expression is elevated in a subset of Col1A2-Cre-CAFs (Fig. 1B). Providing a clinical context to our observations, higher levels CAF-specific *CCN1* score was associated with progressive disease and poor overall survival in patients on anti-PD1 ICIs (Fig. 7B; Table 1). Paralleling these observations, real-time PCR of *Ccn1-*deficient mouse fibroblasts and skin revealed that loss of CCN1 expression resulted in decreased mRNA expression of *Mmp-9* (Fig. 7C and D; in cells, 0.870 ± 0.37 vs. 0.259 ± 0.139; in tissue 1.26 ± 0.109 vs. 0.757 ± 0.107), a gene whose expression is associated with acquisition of anti-PD1 resistance in melanoma (59–61).

**TABLE 1:** Multivariate analysis of CAF-specific *CCN1* score as a prognostic marker for reduced overall survival in GSE78220 (36) in patients on anti-PD1 checkpoint inhibitors

	Overall survival Multivariate analysis	
Characteristic	HR (95% CI)	*P*-value
CAF-specific *CCN1* score	1.39 (1.13–1.70)	**0.0017**
Age	1.06 (0.98–1.15)	0.15
Sex (vs. female)	1.41 (0.18–11.05)	0.74
Previous MAPK inhibitor (vs. none)	5.38 (0.99–29.31)	0.05
*BRAF* mutation	0.47 (0.05–4.43)	0.51
*NRAS* mutation	0.33 (0.04–2.72)	0.30
*NF1* mutation	1.77 (0.28–11.09)	0.54

Collectively, our observations that mice deleted for *Ccn1* in Col1A2-Cre-(universal) CAFs showed defects in ECM elaboration, angiogenesis, and metastasis, taken in context with our bioinformatic analysis of patient-derived data, implicate an essential pathogenic role for CAF-specific expression of *CCN1* in the tumor microenvironment.

## Discussion

Although drugs have been discovered that can retard melanoma progression, patients develop resistance to these regimens. Recently, it has been proposed that drugs targeting either ECM deposition/mechanotransduction or angiogenesis may be beneficial in overcoming this drug resistance and therefore could be effective in combination therapies (10, 11, 17, 18). Accordingly, identification of drug targets that mediate both fibrosis and angiogenesis are of potential interest. In this article, we show, for the first time in any cancer model, that CCN1 is required for both neovascularization and ECM elaboration. Loss of *Ccn1* expression by fibroblasts significantly impaired metastasis of tumor cells to lung and increased recruitment of CD4^+^ T cells into tumors. Our data are of potential translational relevance as, validating our results using an animal model, patient-derived data revealed, for the first time in any cancer, that CAF-specific CCN1 scores are correlated with the expression of both stroma and angiogenic genes and also progressive disease in the context of resistance to an ICI. Finally, we identify the importance of Col1A2-Cre-fibroblast (i.e., universal fibroblast) expression of *Ccn1* in coordinating these activities, emphasizing the importance of the CAF in the phenotype and development of cancers, including melanoma (62–64). Indeed, our conclusion that CCN1 is a novel therapeutic target in melanoma is supported by several lines of patient-derived and animal model-derived data. Our data are consistent with a report demonstrating that CCN1 is induced in response to stiffness in endothelial cells and that knockout of *Ccn1* in endothelial cells inhibits melanoma cancer cell binding to the blood vessels, a critical step in metastasis (65) and that expression of CCN1, downstream of YAP1, may mediate melanoma invasion (66).

Our data support the utility of a novel phenotypic artificial intelligence–based digital pathology image analysis platform to assess deposition and remodeling of tumor stroma in a cancer model. This analysis tool may permit identification of novel therapeutic targets or drugs aimed at remodeling cancer-associated fibrosis. The relevance of the phenotypic approach to quantify fibrosis with FibroNest has also been established in the context of different etiologies of fibrosis in liver (67), and is particularly relevant to this study as we are particularly interested in quantifying the histologic phenotype of fibrosis and its remodeling.

An emerging concept is that alterations in the tumor microenvironment promote invasion and metastasis and thus that targeting these alterations may result in effective anticancer therapies (55, 68). Our article suggests that targeting CCN1 may be an appropriate approach. No drug currently exists in development that specifically targets CCN1; however, our data are consistent with an emerging hypothesis based on data from several laboratories that suggest blocking CCN1 activity, as CCN1 promotes angiogenesis and ECM alignment may be useful alone, or in a combination therapy with either BRAF or ICIs, to treat melanoma. Our data are also consistent with prior data demonstrating that, in breast cancer, a linear “tumor-associated collagen signature” was associated with poor disease-specific and disease-free survival and invasiveness (69, 70).

We are aware of limitations of the *in vivo* melanoma model used in our study, namely that it is known to be poorly immunogenic (56–59). In addition, B16F10 melanoma cells do not express PD-1 (71), precluding the exploration of anti-PD1 antibodies in our studies. That said, it has been proposed that the B16F10/C57BL6 model is useful to study patients resistant to ICIs (72). Thus, the model used is suitable for this study as we are interested in examining the interplay of Col1A2-Cre-fibroblasts with the tumor environment and, specifically, the role of the matricellular protein CCN1 in coordinating this process, including its potential role in ICI resistance. Our data are consistent with recent observations linking CCN1 expression to drug resistance in pancreatic cancers (73).

Finally, it is perhaps somewhat of a surprise that YAP1 nuclear localization and myofibroblast differentiation are observed in the absence of CCN1 expression by fibroblasts and a disorganized collagen network. However, in the absence of a functional collagen network that shields fibroblasts from mechanical stress, resident fibroblasts are mechanically loaded, initiating the tissue repair program including the simulation of adhesive signaling pathways (74–76) and (proto)myofibroblast differentiation (77–82). A likely explanation of the data presented in our article is that CCN1 acts downstream of initial myofibroblast differentiation. Previously, we found that loss of CCN1 by fibroblasts resulted in resistance to increased collagen deposition, but not myofibroblast differentiation, in the bleomycin-induced model of skin fibrosis, concomitant with reduced prolyl-4-hydroxylase and PLOD2 mRNA expression and PLOD2-generated collagen cross-links in CCN1-deficient skin (29). Thus, whereas the related matricellular protein CCN2 is necessary for fibroblast plasticity and myofibroblast differentiation in fibrotic and cancer models (33–35), CCN1 appears to be involved with ECM production/stabilization (this article; ref. 29). That said, it is possible that CCN1 secretion by other cell types may promote myofibroblast formation. However, these results indicate a combination CCN1/CCN2 therapy, possibly based on using the antifibrotic CCN member CCN3, may be useful to treat melanoma (83, 84). Interestingly, although we were able to detect reduced expression of the collagen-binding integrin alpha 11 (85) in CCN2-deficient fibroblasts (34), our approaches examining mRNA expression of CCN1-deficient skin or fibroblasts did not indicate reduced expression of collagen-binding integrins, but rather of collagen cross-linking enzymes (29), providing additional evidence that the roles of CCN2 and CCN1 differ.

In summary, the data presented in this article collectively suggest that Col1A2-Cre-(universal)-fibroblasts, through the production of CCN1, are essential for coordinating ECM deposition and neoangiogenesis in the tumor stroma microenvironment and are consistent with the notion that targeting CAFs, and, in particular, matricellular proteins such as CCN1 (20, 86), represents a novel therapeutic target for melanoma.

## Supplementary Material

Video S1 CCN1 +/+ stroma vasculature microct

Video S2 CCN1 +/+ stroma vasculature microct

Video S3 CCN1 +/+ stroma vasculature microct

Video S4 CCN1 deficient stroma vasculature microct

Video S5 CCN1 deficient stroma vasculature microct

Video S6 CCN1 deficient stroma vasculature microct
